# Differential associations of sex and age with changes in HRQoL during outpatient cardiac rehabilitation

**DOI:** 10.1186/s41687-024-00688-x

**Published:** 2024-01-23

**Authors:** Lorenza L. S. Lanini, Sebastian Euler, Claudia Zuccarella-Hackl, Rubén Fuentes Artiles, David Niederseer, Bianca Auschra, Roland von Känel, Lena Jellestad

**Affiliations:** 1 Faculty of Medicine, University Hospital Zurich, University of Zurich, Zurich, Switzerland; 2 Department of Consultation-Liaison Psychiatry and Psychosomatic Medicine, University Hospital Zurich, University of Zurich, Zurich, Switzerland; 3 Department of Medicine, Limmattal Hospital, Schlieren, Switzerland; 4 Department of Cardiology, University Heart Center, University Hospital Zurich, University of Zurich, Zurich, Switzerland

**Keywords:** Health-related quality of life, Cardiac rehabilitation, Sex, Age

## Abstract

**Introduction:**

Cardiovascular diseases (CVD) represent the world’s leading cause of death. Health-related quality of life (HRQoL) is a widely applied concept of patients’ perceived health and is directly linked to CVD morbidity, mortality, and re-hospitalization rates. Cardiac rehabilitation (CR) improves both cardiovascular outcomes and HRQoL. Regrettably, CR is still underutilized, especially in subgroups like women and elderly patients. The aim of our study was to investigate the predictive potential of sex and age on change of HRQoL throughout outpatient CR.

**Methods:**

497 patients of outpatient CR were retrospectively assessed from August 2015 to September 2019 at the University Hospital Zurich. A final sample of 153 individuals with full HRQoL data both at CR entry and discharge was analyzed. HRQoL was measured using the 36-Item Short Form Survey (SF-36) with its physical (PCS) and mental (MCS) component scale. In two-factorial analyses of variance, we analyzed sex- and age-specific changes in HRQoL scores throughout CR, adjusting for psychosocial and clinical characteristics. Age was grouped into participants over and under the age of 65.

**Results:**

In both sexes, mean scores of physical HRQoL improved significantly during CR (*p* <.001), while mean scores of mental HRQoL improved significantly in men only (*p* =.003). Women under the age of 65 had significantly greater physical HRQoL improvements throughout CR, compared with men under 65 (*p* =.043) and women over 65 years of age (*p* =.014). Sex and age did not predict changes in mental HRQoL throughout CR.

**Conclusions:**

Younger women in particular benefit from CR with regard to their physical HRQoL. Among older participants, women report equal improvements of physical HRQoL than men. Our results indicate that sex- and age-related aspects of HRQoL outcomes should be considered in CR.

**Supplementary Information:**

The online version contains supplementary material available at 10.1186/s41687-024-00688-x.

## Introduction

According to the World Health Organization (WHO), cardiovascular diseases (CVD) continue to represent the world’s leading cause of death [1] in both men and women. Estimates suggest 17.9 million lives lost per year (representing 32% of global mortality), with one third of these deaths prematurely occurring in people under 70 years of age. CVD are, thus, not only a major public health burden, but also strongly affect a patients’ quality of life. Health-related quality of life (HRQoL) a widely applied, multidimensional concept has hereby proven to be a reliable measure of patients’ perceived health [2, 3]. Over the last few years, evidence on an association between poor HRQoL and worse CVD outcome has emerged [4], with poor HRQoL now being recognized as an independent predictor for higher morbidity, mortality, and re-hospitalization rates [5]. In recent years, HRQoL has thus become widely established as an important outcome measure in cardiac care and secondary prevention [6].

In women, CVD is under-recognized due to often atypical clinical presentations, leading to disadvantages in primary and secondary prevention and CVD outcomes [7]. Unfortunately, women with CVD are not only more susceptible to psychosocial stress [8, 9] and worse mental [10] and social [11] health than men, but also show lower HRQoL both at admission to cardiac rehabilitation (CR) and at follow-up [10, 12, 13].

Hence, due attention should be directed to secondary prevention of CVD to positively influence cardiovascular outcome. Considerations of traditional risk factors create the basis for prevention of CVD, yet the impact of sex- [14] and age- [13] specific aspects on CVD outcome is increasingly recognized. Unfortunately, gender gaps still exist disadvantaging women in cardiac care and secondary prevention leading to unfavorable cardiovascular outcomes in women [15]. Women are underrepresented in cardiac research and results are often generalized and not reported in a gender-specific way. As a result, international guidelines and clinical practice are still biased towards men due to a lack of knowledge about sex-specific aspects [16].

As a key element of secondary prevention [17, 18], CR is strongly recommended by international guidelines. CR not only positively influences cardiovascular outcomes [19, 20], but also improves psychosocial distress and HRQoL [21–23], the effect still being present in a longer-term follow-up [24]. Notwithstanding the fact that CR is still underutilized [25, 26], especially in women [27–29], who show greater improvement in mortality rate after CR and greater treatment adherence compared to men [30–32]. Barriers to CR referral have also been reported for elderly patients [33], particularly women [34]. In view of the beneficial effects of CR also in the elderly [13, 35], this is critical and the early targeting of sex- and age-specific [10, 32] needs is crucial. The assessment of potentially relevant psychosocial factors, complementary to modifying traditional risk factors, may allow optimized referral [36] to CR for vulnerable subgroups improving secondary prevention effects and the overall outcome of CVD.

To date, there is only minimal evidence concerning sex- and age- dependent differences in HRQoL in CR patients [10, 12, 13], and the overall outcome is inconclusive. The aim of our monocentric, observational, retrospective study was, thus, to further elucidate the predictive potential of sex and age on change of HRQoL scores in outpatient CR, adjusting for potentially relevant psychosocial and clinical aspects.

## Methods

### Study design and participants

In this monocentric, observational, retrospective study, we assessed data of 497 potentially eligible participants of outpatient CR at the University Hospital Zurich from August 2015 to September 2019. The standardized outpatient CR program comprises 12 weeks of strength and endurance training in 34 sessions of 90 min each. HRQoL assessments were conducted at CR entry and discharge. We included all CR participants, who completed the CR program, had full HRQoL data at both measurement points and who gave written informed consent for the use of their health-related data for research with admission to CR. The study was approved by the Ethics committee of Canton Zurich, Switzerland (REQ-2020-0047).

### Measures

#### Health-related quality of life (HRQoL)

HRQoL was assessed at CR entry and discharge using the 36-Item Short Form Survey (SF-36) with norm-based scores. The SF-36 is a highly reliable [37], established and broadly employed HRQoL questionnaire measuring patients’ perceived emotional and physical health [38]. The SF-36 comprises 36 questions evaluating eight different state-of-health dimensions (vitality, physical functioning, bodily pain, general health perceptions, role limitations in the sense of interference with normal activities due to physical health problems, role limitations due to personal or emotional problems, emotional well-being and social functioning). Those dimensions are summarized into two health component scales: the physical (PCS) and mental component summary scale (MCS) [39]. Scores in each domain range from a minimum of 0 (poor HRQoL) to a maximum of 100 (high HRQoL).

#### Statistical analyses

Statistical analyses were conducted using R version 4.2.0 [40]. The primary outcome of interest was the change in physical and mental HRQoL scores (PCS, MCS) from CR entry to discharge.

First, we performed a preliminary one-way analysis of variance (ANOVA) to detect disease-related, independent variables other than sex with significant impact on physical and mental HRQoL. We used one-way ANOVA and paired-sample t-tests to assess sex-related baseline characteristics as well as to calculate the between-group differences on HRQoL subdomains. We assessed the predictive potential of sex and age (grouped +/- 65 years of age) on SF-36 HRQoL change scores between entry and discharge from CR by means of analysis of variance, using sex as grouping variable. Since sex-specific effects for different age groups were reported [41], we included an interaction term *sex x age-group* in our analyses. Cases with incomplete data were excluded from our analysis.

Based on previous research and clinical experience, we controlled for the following psychosocial and disease-related covariates: housing situation (living alone vs. living with others), presence of a psychiatric disorder, change in 6-minute-walk distance (calculated using the difference between the walk distance at CR entry and discharge), ischemic heart disease and modifiable traditional cardiovascular risk factors (hypertension, dyslipidemia, overweight, smoking and type II diabetes). Social support (assessed with housing situation as a proxy) is reportedly associated with higher psychological well-being and prevention of emotional distress after cardiac disease [42, 43], while the presence of a psychiatric disorder impacts negatively on CR outcomes [44]. With regard to the 6MWD, better 6MWD performance has been linked to greater HRQoL improvements [13, 41].

## Results

### Baseline clinical characteristics

**Fig. 1:**
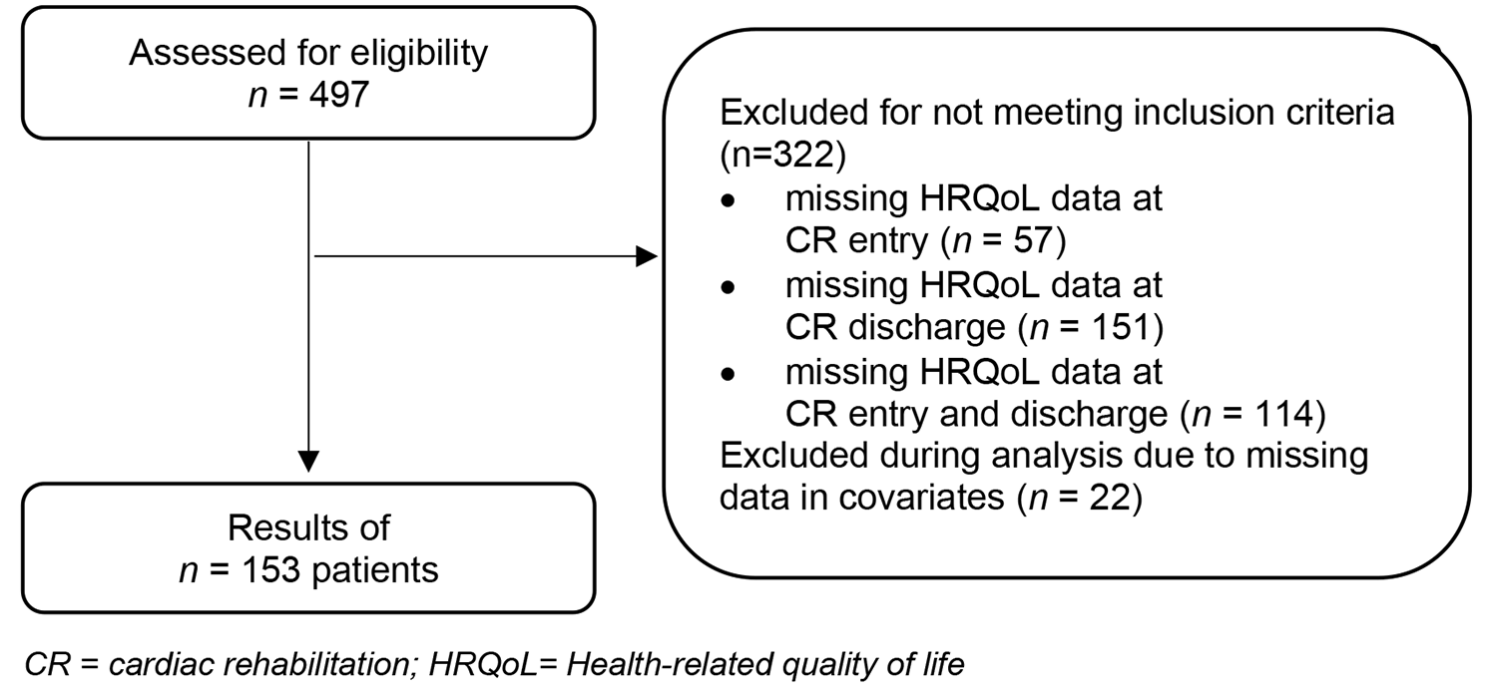
Flowchart of CR participants. (CR = cardiac rehabilitation; HRQoL = Health-related quality of life)

A total of *n* = 153 individuals with complete data including HRQoL data at both CR entry and discharge were analyzed (Fig. 1). Of the excluded patients *n* = 57 had missing data at CR entry, *n* = 151 had missing data at CR discharge and *n* = 114 had missing HRQoL data at both measurement points. Baseline characteristics of the 153 study participants are displayed in Table 1. More than four fifth where male, with an average age of 59.53 (SD = 12.21) years. Female patients were older at CR admission than their male counterparts. In both sexes, over two thirds lived in a household of two or more persons. Comorbid psychiatric disorders were rare (< 15% of the sample).

**Table 1:** Clinical characteristics of 153 study participants

	Total	Men	Women
*N*	153	131 (85.6%)	22 (14.4%)
Age (years)^1^	60.44 (12.05)	59.53 (12.21)	65.86 (9.56)
Over 65	55 (36%)	42 (32.1%)	13 (59.1%)
Housing conditions			
living alone	46 (30%)	39 (30%)	7 (32%)
household ≥ 2	107 (69.9%)	92 (70.2%)	15 (68.2%)
Change in 6MWD (m)^1^	47.25 (53.44)	46.36 (53.62)	52.59 (53.24)
Presence of ICD-10 diagnosis			
ischemic heart disease	124 (81%)	109 (83%)	15 (68%)
hypertension	87 (57%)	79 (60%)	8 (36%)
dyslipidemia	90 (59%)	79 (61%)	11 (50%)
overweight	34 (22%)	27 (21%)	7 (32%)
smoking	79 (52%)	71 (54%)	8 (36%)
diabetes mellitus	24 (16%)	19 (15%)	5 (23%)
positive family history for CVD	45 (30%)	35 (27%)	10 (45%)
Psychiatric disorder	18 (12%)	17 (13%)	1 (9%)

Descriptives. CVD = cardiovascular disease; m = meters; ^1^Mean (SD); 6MWD = 6-minute walk distance

The 153 included patients did not differ significantly from the 344 excluded participants in terms of age and sex. (Table 1, Appendix).

### HRQoL mean scores with entry to and discharge from CR

In paired-sample t-tests both sexes improved significantly in their physical HRQoL throughout CR (*p* <.001). However, with regard to mental HRQoL, significant improvements were observed in men only (*p* =.003). Women had significantly lower physical HRQoL than men at entry to CR, but not with discharge from CR. Mental HRQoL did not differ between sexes, neither at entry to, nor at discharge from CR. The mean scores of physical and mental HRQoL at CR entry and discharge CR are displayed in Table 2.

**Table 2:** Means of mental and physical HRQoL subdomains at entry in and discharge of CR

	Total	Men	Women	*p*
Physical HRQoL				
CR entry	41 (9.95)	41.86 (9.54)	35.87 (10.98)	0.008*
CR discharge	47.41 (9.27)	47.96 (9.24)	44.15 (8.94)	0.069
*p*		< 0.001*	< 0.001*	
Mental HRQoL				
CR entry	48.74 (11)	48.64 (10.92)	49.29 (11.69)	0.829
CR discharge	51.07 (9.54)	51.08 (9.42)	51.01 (10.47)	0.955
*p*		0.003*	0.407	

T-tests and one-way ANOVA with means M (SD) of HRQoL scores (as assessed with the SF-36). CR = cardiac rehabilitation; HRQoL = Health-related quality of life; *= significant difference at α = 0.05

### Predictors of HRQoL

In two-factorial analyses of variance, associations between gender, age, psychosocial and disease-related variables and the change in HRQoL were calculated. The overall model explained 50.4% of the variance (η² = 0.504) for physical HRQoL and 41.1% of the variance (η² = 0.411) for mental HRQoL (Table 3).

**Table 3:** Results of two-factorial analyses of variance on changes of mental and physical HRQoL

	Physical HRQoL		Mental HRQoL	
	*F*	*p*	*F*	*p*
Gender (male)	0.071	0.791	3.09	0.081
Age (years)				
over 65	0.787	0.377	0.013	0.91
Housing conditions				
living alone	0.518	0.473	0.112	0.738
Change in 6MWD (m)	1.445	0.180	0.966	0.328
Presence of ICD-10 diagnosis				
ischemic heart disease	5.451	0.021*	0.001	0.97
hypertension	0.873	0.352	0.012	0.914
dyslipidemia	0.702	0.404	0.003	0.959
overweight	2.913	0.09	0.38	0.538
smoking	0.004	0.95	1.042	0.309
diabetes mellitus	5.882	0.017*	1.556	0.213
psychiatric disorder	9.23	< 0.01*	4.382	0.038*
Interaction term				
sex x age-group^1^	4.221	0.042*	0.038	0.844
R2	0.504		0.411	

Two-factorial analyses of variance with unstandardized coefficients. HRQoL = Health-related quality of life; m = meters; 6MWD = 6-minute walk distance; *p <.05; ^1^age grouped as +/-65 old

#### Sex and age as predictors of HRQoL

**Fig. 2:**
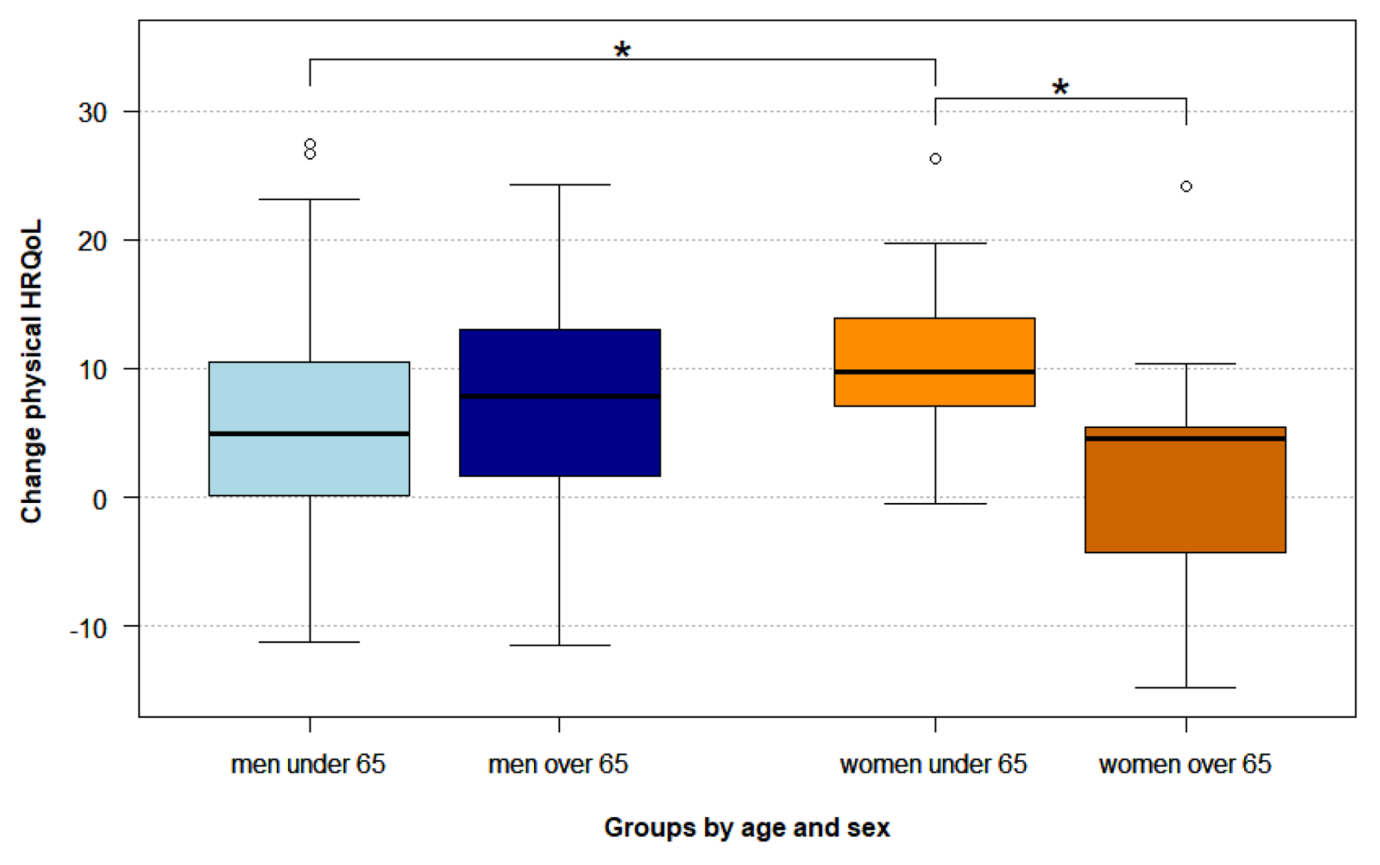
Changes of physical HRQoL scores by subgroups as investigated in post-hoc analysis. (*p <.05)

In analyses of variance (Table 3) on changes in physical HRQoL, main effects of sex and age (grouped +/- 65 years) were not significant. However, the interaction sex*age-group proved to be statistically significant (*F*(1,128) = 4.22, *p* =.042). We performed a post-hoc analysis (Bonferroni-Holm) controlling for multiple testing to differentially assess the predictive potential of the 4 groups (women over/under 65 and men over/under 65 years of age). Being female and below 65 years of age predicted the greatest improvements of physical HRQoL throughout CR, compared to women over 65 (*p* =.014), and men under 65 (*p* =.043). Figure 2 displays the changes of physical HRQoL scores by subgroups, as revealed in post-hoc analysis.

With regard to mental HRQoL, sex, age (grouped +/- 65 years) and the interaction sex* age-group showed no significant association with mental HRQoL changes throughout CR.

## Discussion

This monocentric observational, retrospective study on 153 participants in outpatient CR examined the predictive potential of sex and age on changes of HRQoL throughout CR.

CR improved mean scores of physical HRQoL in both sexes, those of mental HRQoL only in men.

With regard to physical HRQoL, we found younger women under 65 years of age to particularly benefit from CR with regard to their physical HRQoL, Older women, however, showed the same improvements in physical HRQoL as older men. With regard to mental HRQoL, older age predicted greater improvements throughout CR, while sex and its interaction with age revealed no predictive potential on mental HRQoL change.

Current evidence on sex- and age-related differences of HRQoL in cardiac patients is scarce. A few small scale studies show poorer HRQoL in women with CVD after short-term follow-up [45–48]. In CR, women report poorer mental health (anxiety and depression) at CR entry compared to men [49]. Specific sex- and age-related effects of CR on HRQoL outcomes are poorly explored. Our results confirm previous findings of poorer physical and mental HRQoL [10, 12] in women compared to men at entry to outpatient CR and poorer emotional and physical HRQoL in women compared to men at entry to inpatient CR [13]. The greater improvement of physical HRQoL we observed in younger women compared with younger men is in agreement with comparable recent findings [13], and in disagreement with others [10]. These divergent results might be partially explained by the differing sample sizes [13] and patient characteristics, e.g. higher proportion of women in the total sample [10, 12, 13]. This significantly higher improvement in physical HRQoL of younger women compared with men throughout CR is, however, to be particularly emphasized. Physical activity positively impacts HRQoL [50], with women benefiting particularly from tailored CR programs [51]. Concurrently, women and younger participants especially benefit from CR by improving their exercise capacity [52]. Physical inactivity at the same time represents a recognized cardiovascular risk factor [53]; furthermore, the positive effects of physical activity on CVD [54] have already been documented and should be properly addressed, improving secondary as well as primary cardiac prevention. On the one hand, the lack of activity and its negative impacts on HRQoL especially in women should be highlighted, particularly considering the benefit CR has on improving their physical HRQoL. On the other hand, the importance of CR referral should particularly be emphasized for women given the described sex-disparity, thus encouraging physical activity in women.

With regard to mental HRQoL, our results show no significant associations between age and sex on mental HRQoL changes throughout CR. This is in contrast to previous evidence of greater mental HRQoL improvements in elderly patients (> 75 years) [13]. The fact that we did not see these effects could have been due to our smaller sample size. Also, the different grouping of age in the aforementioned and our study may have contributed to these discrepant findings.

In our study, we controlled for the social factor of living alone versus not living alone as a proxy of social support, as well as for the presence of a comorbid psychiatric disorder. Social support has been reported as a relevant and protecting factor for both incidence and prognosis of CVD [55]. Our results did not reveal any difference in HRQoL change scores between participants living alone vs. those not living alone. This may be attributable to our moderate sample size, the predominance of participants in our sample not living alone, as well as not being able to take into account the social support experienced outside one’s household. A three-dimensional questionnaire with physical, mental as well as social aspects of quality of life may be more appropriate to tackle this question [13]. It is also worth noting that our traditional sex-driven constructs may influence the extent and perception of support, affecting women who experience less support on the one hand [56] and struggle to seek it on the other. This may be attributable to the fact that they do not want to burden others or are afraid of not being taken seriously [57]. Interestingly, in a recent systematic review additional psychosocial strategies seem to positively impact on women’s CR outcomes compared to traditional CR [51]. Other strategies in addressing sex-specific needs include women-only CR programs, however, results are inconsistent. While our results suggest that women benefit from traditional CR, there are grounds for considering that tailored CR programs could further improve CR outcomes in women. This again points to the fact that sex-specific and psychosocial aspects relevant to women are still not sufficiently addressed in secondary prevention and CR.

Future larger studies are needed to further focus on these complex interplays between sex, age, psychosocial aspects and CR outcomes to expand knowledge and improve secondary prevention also in vulnerable patient groups.

With regard to psychiatric disorders, CR has been reported to improve mental health outcomes like anxiety and depression [58]. Also, recent evidence found the probability of potentially eligible patients participating in CR to be greater in patients with a psychiatric disorder [59]. Our results hint at psychiatric disorders being associated with lower gain in physical and mental HRQoL during CR.

## Strengths and limitations

Given the uniformity of the CR program, we benefited from a well-defined and standardized intervention paradigm. We used the widely applied SF-36 survey for HRQoL assessment, which has been used in countless other studies and offers a sound comparability. The retrospective nature of our study without a control group cannot rule out bias due to spontaneous improvements in HRQoL without participation in a CR program, impairing the representativeness of the results.

The moderate sample size, the underrepresentation of women in our sample and a fair proportion of missing HRQoL data limits the power of our study and the generalizability of results. Missing data on CR discharge should be regarded particularly critically in clinical practice, as they reduce the informative value of the effectiveness of CR in this respect. At the same time, the self-evaluation of HRQoL can also be a helpful assessment tool for the patients themselves in order to objectify the progress made during CR. This important tool should therefore not be dispensed with. The small effect size of our model on mental HRQoL change also needs to be recognized, which limits its informative value. Especially with regard to the changes in mental HRQoL, our sample may have been too small to capture predictive effects of sex and age. Since the CR program is primarily aimed at improving physical functioning, the more subtle effects on mental health could only be visible in a larger sample. Future research on this topic in studies with larger samples is recommended. However, the fact that, we were able to reproduce in substantial respects and in an outpatient setting the findings of an earlier, much larger study on sex-related changes in HRQoL with respect to higher gain in physical HRQoL in younger women clearly shows the relevance of this aspect in CR.

## Conclusions

Our findings add to the still meager evidence for sex and age as predictors of HRQoL outcomes in CR. We provide important insights calling for special attention to younger women, who, although having significantly lower physical HRQoL mean scores than men at CR entry, benefit the most from CR in this regard. Among older patients above 65 years of age, women show similar improvements in their physical HRQoL. This observation underscores that the low CR referral and participation rates in women are particularly problematic and need to be focused on in future efforts for optimal cardiovascular prevention. In summary, a better understanding of sex- and age related aspects of HRQoL improvements throughout CR is crucial to allow offsetting substantial disadvantages in vulnerable patient groups and the development of better tailored CR programs in the future.

### Electronic supplementary material

Below is the link to the electronic supplementary material.

Supplementary Material 1

## Data Availability

The employed data cannot be publicly disclosed due to privacy and ethical restrictions. The data can be provided upon reasonable request from corresponding author.

## Abbreviations

ANOVA: Analysis Of Variance
CR: Cardiac Rehabilitation
CVD: Cardiovascular Diseases
HRQoL: Health Related Quality of Life
MCS: Mental Component Scale
PCS: Physical Component Scale
SD: Standard Deviation
SF-36: 36-Item Short Form Survey
WHO: World Health Organization
6MWD: 6-Minute-Walk Distance
