# Supplementary material for: Differential associations of sex and age with changes in HRQoL during outpatient cardiac rehabilitation
Source: J Patient Rep Outcomes. 2024 Jan 23;8:11. doi: 10.1186/s41687-024-00688-x (PMC10805744; doi:10.1186/s41687-024-00688-x)
Supplement: Supplementary file 1 — Supplementary Material 1 [file 41687_2024_688_MOESM1_ESM.docx]

# Appendix

**Table 1. One-way ANOVA to test risk of bias**

|  | **Included Patients (*n*)** | **Excluded Patients (*n*)** | **(df1, df2) F** | ***p*** |
| --- | --- | --- | --- | --- |
| Sex (male) | 153 | 344 | (1, 495) 3.679 | 0.56 |
| Age | 153 | 343 | (1, 494) 0.890 | .346 |
| Over 65 | 153 | 343 | (1, 494) 3.087 | 0.80 |
